# Menu Labeling and Calories Purchased in Restaurants in a US National Fast Food Chain

**DOI:** 10.1001/jamanetworkopen.2023.46851

**Published:** 2023-12-15

**Authors:** Pasquale E. Rummo, Tod Mijanovich, Erilia Wu, Lloyd Heng, Emil Hafeez, Marie A. Bragg, Simon A. Jones, Beth C. Weitzman, Brian Elbel

**Affiliations:** 1 Department of Population Health, New York University Grossman School of Medicine, New York; 2 Steinhardt School of Culture, Education, and Human Development, New York University, New York; 3 Wagner Graduate School of Public Service, New York University, New York

## Abstract

**Importance:**

Menu labeling has been implemented in restaurants in some US jurisdictions as early as 2008, but the extent to which menu labeling is associated with calories purchased is unclear.

**Objective:**

To estimate the association of menu labeling with calories and nutrients purchased and assess geographic variation in results.

**Design, Setting, and Participants:**

A cohort study was conducted with a quasi-experimental design using actual transaction data from Taco Bell restaurants from calendar years 2007 to 2014 US restaurants with menu labeling matched to comparison restaurants using synthetic control methods. Data were analyzed from May to October 2023.

**Exposure:**

Menu labeling policies in 6 US jurisdictions.

**Main Outcomes and Measures:**

The primary outcome was calories per transaction. Secondary outcomes included total and saturated fat, carbohydrates, protein, sugar, fiber, and sodium.

**Results:**

The final sample included 2329 restaurants, with menu labeling in 474 (31 468 restaurant-month observations). Most restaurants (94.3%) were located in California. Difference-in-differences model results indicated that customers purchased 24.7 (95% CI, 23.6-25.7) fewer calories per transaction from restaurants in the menu labeling group in the 3- to 24-month follow-up period vs the comparison group, including 21.9 (95% CI, 20.9-22.9) fewer calories in the 3- to 12-month follow-up period and 25.0 (95% CI, 24.0-26.1) fewer calories in the 13- to 24-month follow-up period. Changes in the nutrient content of transactions were consistent with calorie estimates. Findings in California were similar to overall estimates in magnitude and direction; yet, among restaurants outside of California, no association was observed in the 3- to 24-month period. The outcome of menu labeling also differed by item category and time of day, with a larger decrease in the number of tacos vs other items purchased and a larger decrease in calories purchased during breakfast vs other times of the day in the 3- to 24-month period.

**Conclusions and Relevance:**

In this quasi-experimental cohort study, fewer calories were purchased in restaurants with calorie labels compared with those with no labels, suggesting that consumers are sensitive to calorie information on menu boards, although associations differed by location.

## Key Points

**Question:**

Is providing nutrition information on menus associated with calories purchased in a national fast food restaurant chain?

**Findings:**

In this cohort study of 2329 Mexican-inspired fast food restaurants in 6 US locations, menu labeling was associated with 25 fewer calories purchased per transaction in the first 2 years after implementation compared with those with no labels. Findings were similar over time, with 22 fewer calories purchased in the 3- to 12-month follow-up period and 25 fewer calories purchased in the 13- to 24-month follow-up period.

**Meaning:**

Menu labeling was linked to fewer calories purchased over time compared with no calorie information on menu boards; these findings suggest that, in combination with other food labeling and population-level policies, menu labeling may be associated with decreased daily caloric intake.

## Teaser

This cohort study examines whether labeling menus with nutrition information is associated with calories purchased at a US fast food chain.

## Introduction

Calorie labeling is a population-level strategy designed to reduce unhealthy food purchases by providing nutrition information at the point of selection in retail food environments, with the goal of supporting customers’ ability to accurately evaluate the healthfulness of foods and beverages.^1^ The information provided by menu labels may thus help people choose lower-calorie meals in restaurant settings and/or encourage retailers to offer lower-calorie items.^2^ This strategy may yield a reduction in daily caloric intake and create a positive influence on population-level health outcomes, including obesity and cardiovascular disease.^3^

Menu labeling became federal policy as part of the Patient Protection and Affordable Care Act of 2010, requiring that chain restaurants with 20 or more locations list the caloric value of standard items on menu boards by May 2018.^4^ Before national rollout, menu labeling requirements were proposed in more than 40 municipalities and states and implemented in 11 of those areas.^5^ Previous studies have sought to evaluate the association between menu labeling legislation and calories and nutrients purchased in restaurant settings, with mixed results.^6^ Some studies reported that menu labeling led to a small decrease in the calories purchased,^7,8,9,10,11,12^ while other studies found no association,^13,14,15,16,17,18,19,20^ with substantial variation in results by restaurant type,^21^ food item category,^8^ and age group.^22^

Most prior work has used survey data and lacked sufficient power to detect small changes in calories purchased.^7,15,16,19,23,24^ A few evaluations have used sales data from chain restaurants,^8,10,11,25^ but this work has several limitations, including limitations in causal inference and generalizability. For example, Petimar et al^10,11^ lacked a comparison group when they used sales data from 3 restaurant chains in Louisiana, Mississippi, and Texas. They found that menu labeling was associated with small decreases in mean calories purchased after voluntary franchise labeling and nationally mandated labeling, but their assessment could not account for natural variation in sales over time or unrelated events that may have affected purchase decisions. Two other studies used sales data to assess menu labeling laws in New York City and King County, Washington, including legislation requiring calorie information on drive-through menu boards, and found small changes in calories purchased in New York City and no changes in King County, Washington, with slightly larger changes for nonbeverage items and lunch-time purchases at Starbucks locations in New York City.^8,25^ Geographic coverage in these studies, however, was limited and results may not be generalizable to other locations.

We sought to address these limitations by using 9 years of transaction-level sales data from 1280 restaurants in a national chain, including data from restaurants in locations where menu labeling was implemented before the national rollout and comparison restaurants in which labeling was never implemented. Using a quasi-experimental design and a difference-in-differences model in a cohort study, we examined menu labeling and calories purchased per transaction and assessed potential differences by location, food item category, and time of day.

## Methods

### Data Sources

We acquired data from 10 575 Taco Bell restaurants in the US, including all unique restaurants that opened and closed during the study period, representing 5.33 billion transactions from calendar years 2007 to 2014. Transaction data included the name, number, and price of items purchased, including customizations; the date, time, and location of the purchase; how the order was placed (drive-through, eat-in, and takeout); and the type and size of beverages in drive-through transactions. Menus are standard, so menu offerings do not vary across restaurant locations. Because we could not assign calories to self-serve beverage purchases in eat-in and takeout transactions, we excluded beverage data from in-store transactions. Per the Common Rule (45 CFR §46), institutional review board approval was not sought because this study is not human participant research. Reporting followed the Strengthening the Reporting of Observational Studies in Epidemiology (STROBE) reporting guideline for cohort studies.

Between 2007 and 2012, several localities mandated and implemented labeling, including several states (California, Maine, and Vermont), counties (King County, Washington; Montgomery County, Maryland; Albany County, New York; Schenectady County, New York; Suffolk County, New York; Ulster County, New York; and Westchester County, New York), and cities (Philadelphia, Pennsylvania; and New York City, New York).^5^ We identified dates of menu labeling implementation using information from the Center for Science in the Public Interest, which tracks such legislative efforts, legislation source text, peer-reviewed literature, news articles, and direct communication with Taco Bell (Table 1). For restaurants in Maine and Vermont; Philadelphia, San Francisco, and New York City; and Albany, Ulster, and Westchester Counties, New York, we either observed incomplete data or no data, due to the staggered rollout of a new point-of-sales system during the implementation of menu labeling.

**Table 1.** Distribution of Restaurants in Menu Labeling Group and Comparison Group by Location

Location	Date of menu labeling implementation		No. (%)		Unique comparison restaurants in analysis, No. (% of eligible comparison)	No.	
	Drive-through	In-store	Unique treated restaurants in analysis^a^	Unique eligible comparison restaurants^b^^,^^c^		Counties, comparison restaurants in analysis	States, comparison restaurants in analysis
King County, WA	Dec 31, 2008	Aug 1, 2008	3 (1.3)	1743 (58.5)	410 (23.5)	139	27
Suffolk County, NY	Oct 28, 2010	Oct 28, 2010	16 (3.4)	1519 (51.0)	519 (34.2)	201	31
Schenectady County, NY	Sept 12, 2010	Sept 12, 2010	1 (0.2)	1468 (49.3)	100 (6.8)	77	24
Montgomery County, MD	Jan 1, 2011	Jan 1, 2011	3 (0.6)	1481 (49.7)	216 (14.6)	101	24
California	May 1, 2018^d^	Jan 1, 2011	450 (94.3)	1855 (44.1)	1512 (81.5)	402	34
Vermont	May 18, 2012	May 18, 2012	1 (0.2)	1313 (62.3)	100 (7.6)	67	21
Overall			477 (100)	2979 (100)	1855 (62.3)	411	34

^a^ Approximately 0.63% of restaurants were excluded from the analytic sample because of a lack of data available in the relevant baseline period (ie, 3-8 months before menu labeling implementation).

^b^ Restaurants in which labeling was never implemented before national implementation (n = 5721) were considered eligible for inclusion in the comparison group before matching and weighting. For inclusion, comparison restaurants were required to have monthly transaction data in the baseline period, defined as the 3 to 8 months before the date of location-specific and setting-specific (in-store, drive-through) menu labeling; and were also required to have monthly transaction data in the 24-month follow-up period. In addition, restaurants were required to have complete data on all matching variables.

^c^ The sum of the percentages exceeds 100% because comparison restaurant units can be used more than once (ie, replacement).

^d^ Date of national rollout.

In Montgomery, Suffolk, and Schenectady Counties, and Vermont, the implementation of calorie labeling on in-store menu boards occurred at the same time as implementation on drive-through menu boards (Table 1). In California, restaurants were required to implement labeling on in-store menu boards by January 1, 2011, but the requirement did not extend to drive-through menu boards until national legislation went into effect in 2018; thus, we excluded drive-through data from analyses with California restaurants. In restaurants located in King County, Washington, legislation required the addition of calorie labels to in-store menu boards on December 31, 2008, and to drive-through menu boards on August 1, 2009. We confirmed that calorie information was added to menu boards in accordance with the dates of location-specific legislation. For study inclusion, we required that restaurants have monthly transaction data in the baseline period, which we defined as the 3 to 8 months before the date of location- and setting-specific (in-store, drive-through) menu labeling (n = 474 restaurants) (eTable 1 in Supplement 1).

### Comparison Group

Restaurants in which labeling was never implemented before national implementation (n = 8441) were considered eligible for inclusion in the comparison group. We required that comparison restaurants have at minimum monthly transaction data that matched the data availability of each restaurant in the menu labeling group during the baseline and 24-month follow-up period, and we required that all restaurants have complete data on all matching variables (n = 2979).

We used synthetic control methods to construct a comparison unit for each restaurant in the menu labeling group, using a suite of restaurant- and community-level characteristics (eTable 2 in Supplement 1). Synthetic control methods allow for the construction of a single synthetic comparison unit matched to each treatment unit by calculating a weighted average of the outcome variable from a source of multiple potential comparison units similar to the treated units in the baseline period.^26,27,28^ Thus, each synthetic comparison unit in our study represents a weighted average of data from multiple restaurants in the potential comparison pool, matched by relative month of implementation of menu labeling by location. To optimize computational efficiency, we first restricted the pool of potential comparison restaurants to the 100 most characteristically similar restaurants with Mahalanobis distance matching, which is an approach to pairing units that have similar covariates on a Euclidean distance.

For the 474 restaurants in the menu labeling group, we estimated 474 synthetic control units (with each unit weighted to 1 to match the treatment unit), using the tidysynth package in R, version 4.1.2 (R Foundation for Statistical Computing), which was used for all statistical analyses (eMethods in Supplement 1 provides the code). Restaurant- and community-level characteristics of treatment and comparison groups were similar between treatment and comparison groups after weighting (eTable 2 in Supplement 1). In the final analytic sample, comparison restaurants were located in 878 counties in 35 states (Table 1). eFigure 1 in Supplement 1 presents a map of the geographic locations of comparison restaurants by treatment location.

### Outcomes

Our primary outcome was mean calories per transaction and secondary outcomes included grams of total fat, carbohydrates, protein, saturated fat, sugar, and dietary fiber, and milligrams of sodium. We assigned calories and nutrients to unique menu items (n = 3517) using MenuStat, a nutrition database of foods and beverages served by national chain restaurants.^29^ Using both automated and manual matching of menu items with nutrition information (eMethods in Supplement 1), we matched more than 95% of total purchased items every quarter.

### Statistical Analysis

Data were analyzed from May to October 2023. We excluded data from 2 months before and 2 months after the date of menu labeling implementation to account for variation in the implementation of menu labeling by restaurant owners and variation in awareness of menu labeling among customers. We also dropped 4 restaurant-month observations with implausible values of the primary outcome (<50% of mean calories per transaction per restaurant). We compared the differences in the calories purchased per transaction before and after menu labeling implementation between menu labeling and comparison restaurants across all locations using a 2-way fixed effects regression model, with time modeled as relative month from implementation by location and calendar month fixed effects to control for seasonality (eMethods in Supplement 1 provides the equation).^30^

Given that approximately 94% of the restaurants in our sample were located in California, we assessed heterogeneity by location by stratifying our primary model by California and non-California restaurants. To understand heterogeneity by time of day, we stratified by late night (12:00-3:59 am), breakfast (4:00-10:59 am), lunch (11:00 am-1:59 pm), afternoon (2:00-4:59 pm), dinner (5:00-8:59 pm), and evening (9:00-11:59 pm). To understand potential mechanisms underlying the response to calorie labeling, we analyzed count of items transacted per restaurant-month by item category (taco, burrito, salad, other entree, dessert, and beverage). To understand potential heterogeneity by order setting, we also stratified by in-store and drive-through orders among the sample of non-California restaurants. For all analyses, we estimated the average effect size of menu labeling in the 3 to 24 months following menu labeling, using the mean of the monthly difference-in-differences estimates per period.

To assess whether results were robust to key model specifications, sensitivity analyses included reestimating our primary model using (1) no matching, (2) 10- and 12-month baseline periods, and (3) only restaurants that were open for at least 12, 15, 18, 21, and 24 months following menu labeling implementation (to address potential treatment effect size heterogeneity over time). We assessed whether changes to the number of calories of existing menu items may have contributed to the estimates by comparing mean calories purchased between the baseline and follow-up periods for the treatment and comparison groups for only the 100 highest-selling items sold in both periods, and reestimating our primary model excluding items introduced in either period. We used a 2-sided α level of .05 as the threshold for statistical significance.

## Results

The final sample size was 2329 unique restaurants and 31 468 restaurant-month observations, with 474 restaurants in the menu labeling group and 474 synthetic control units. The eMethods in Supplement 1 reports changes to sample size for each restriction per group. In the baseline period, mean (SD) calories per transaction was 1035 (135) in the menu labeling group and 1056 (137) in the comparison group (Table 2). Taco orders (which could include multiple tacos) represented most sales by item category (50.9%) (eFigure 2 in Supplement 1). Transactions during lunch (34.6%) and dinner (27.8%) represented most sales by time of day (eFigure 3 in Supplement 1); the mean (SD) calories per transaction was highest during dinner (1143 [142]) and evening (1088 [186]).

**Table 2.** Descriptive Statistics of Transactions, Overall and by Location, Item Category, and Time of Day

Variable	Calories, mean (SD)^a^				Percentage sales, %^b^				Count of items sold, mean (SD)^c^			
	Menu labeling		Comparison group		Menu labeling		Comparison group		Menu labeling		Comparison group	
	Baseline^d^	Follow-up^e^	Baseline	Follow-up	Baseline	Follow-up	Baseline	Follow-up	Baseline	Follow-up	Baseline	Follow-up
Overall^f^	1035 (135)	1041 (139)	1036 (132)	1067 (131)	100	100	100	100	61 910 (22 899)	58 147 (22 722)	69 640 (18 153)	66 680 (16 434)
Location												
California^g^	1014 (100)	1021 (110)	1015 (97)	1049 (105)	84.5	84.0	84.2	83.6	59 251 (20 173)	55 696 (20 110)	68 968 (14 541)	65 165 (14 161)
Non-California^h^	1393 (134)	1396 (120)	1394 (132)	1398 (121)	15.5	16.0	15.8	16.4	98 853 (26 317)	95 254 (27 243)	78 972 (43 339)	89 624 (27 600)
Item category												
Taco	220 (6)	214 (7)	222 (5)	216 (6)	50.9	53.7	54.4	57.4	31 036 (11 280)	30 986 (12 001)	37 803 (9111)	38 146 (9276)
Burrito	447 (11)	450 (14)	455 (9)	459 (13)	26.9	25.5	23.2	21.9	16 416 (6159)	14 681 (5831)	16 104 (4530)	14 527 (4026)
Salad	145 (7)	152 (14)	146 (5)	149 (7)	4.1	3	3.6	3	2477 (1442)	1733 (984)	2520 (1017)	1963 (633)
Other entree	476 (34)	485 (30)	472 (36)	487 (31)	13.7	13.8	14.7	14.2	8342 (3549)	7980 (3649)	10 219 (3241)	9418 (2959)
Dessert	203 (17)	214 (20)	203 (9)	214 (16)	2.9	2.5	3.1	2.5	1783 (834)	1457 (834)	2152 (735)	1651 (710)
Beverage^i^	288 (9)	222 (58)	286 (9)	213 (38)	1.5	1.4	1.1	1.1	15 642 (6455)	15 598 (5102)	13 031 (7203)	14 090 (4181)
Time of day^j^												
Late night	685 (1252)	715 (979)	248 (424)	273 (560)	1.3	1.3	1.1	1.1	3345 (6024)	3603 (5953)	511 (1752)	555 (1619)
Breakfast	898 (145)	841 (179)	900 (125)	916 (113)	5.8	6.2	3.9	3.7	1533 (1760)	1415 (2157)	742 (1195)	649 (1126)
Lunch	974 (91)	983 (94)	965 (89)	986 (93)	34.5	33.8	36.9	36.4	10 686 (6031)	9549 (6322)	9779 (4527)	8857 (4205)
Afternoon	970 (111)	984 (125)	976 (101)	1012 (111)	22.8	23.4	21.3	21.9	6656 (5270)	6083 (4938)	5469 (3603)	4759 (2924)
Dinner	1144 (141)	1172 (152)	1147 (131)	1200 (144)	27.8	27.7	28.7	29.2	9148 (7263)	8503 (8585)	8426 (4341)	7586 (3809)
Evening	1089 (185)	1100 (587)	1058 (150)	1099 (156)	8	7.5	8.1	7.7	2769 (4981)	2314 (4662)	2314 (2441)	1822 (2098)

^a^ Mean calories per transaction.

^b^ Percentage of sales contribution in the corresponding period. The raw numbers to accompany all percentages are not available given the proprietary nature of the data.

^c^ Mean count of items sold per restaurant-month.

^d^ Defined as the 3 to 8 months before the date of location-specific and setting-specific menu labeling.

^e^ Defined as the 3 to 12 months after the date of location-specific and setting-specific menu labeling.

^f^ Within-restaurant data only for California, using covariate balancing propensity score matching, and within-restaurant plus drive-through data for other locations, using synthetic control matching.

^g^ Excludes beverage data.

^h^ Includes beverage data from drive-through transactions.

^i^ Drive-through transactions only.

^j^ Late night (12:00-3:59 am), breakfast (4:00-10:59 am), lunch (11:00 am-1:59 pm), afternoon (2:00-4:59 pm), dinner (5:00-8:59 pm), and evening (9:00-11:59 pm).

**Figure 1.**
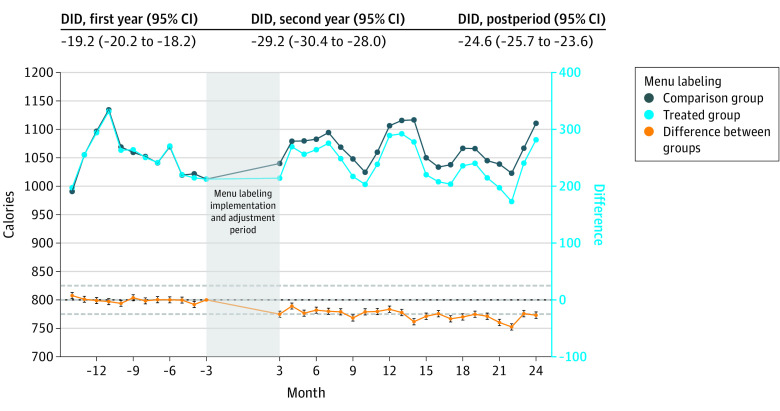
Difference-in-Differences (DID) Estimates of Calories Purchased per Transaction After Implementation of Menu Labeling, Overall

**Figure 2.**
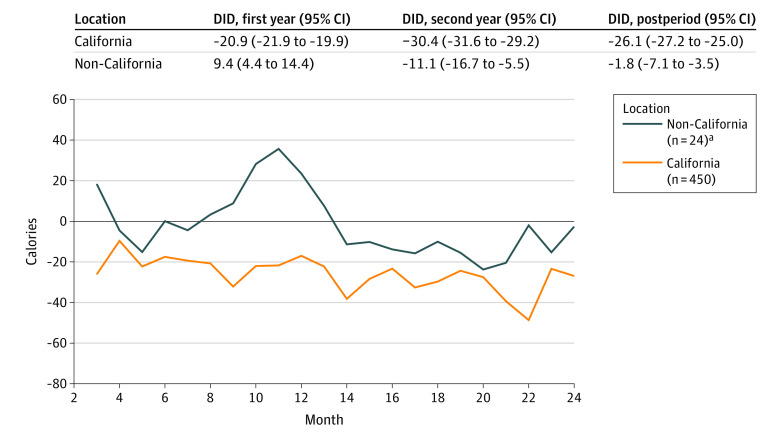
Difference-in-Differences (DID) Estimates of Calories Purchased per Transaction After Implementation of Menu Labeling, by Location ^a^Non-California locations were King County, Washington; Montgomery County, Maryland; Suffolk and Schenectady Counties, New York; and Vermont.

Changes in calories purchased were approximately parallel between menu labeling and comparison groups in the baseline period (Figure 1). Customers purchased 24.7 (95% CI, 25.7-23.6) fewer calories per transaction from restaurants in the menu labeling group in the 3- to 24-month follow-up period vs the comparison group, including 21.9 (95% CI, 20.9-22.9) fewer calories in the 3-month and 25.0 (95% CI, 24.0-26.1) fewer calories in the 13- to 24-month follow-up periods (Table 3; eTable 3 in Supplement 1). Changes in the nutrient content of transactions were consistent with calorie estimates, with small decreases in the amount of each macronutrient and micronutrient purchased in restaurants in the menu labeling group (eTable 4, eFigure 4 in Supplement 1). Findings in California were consistent with our overall estimates, with 26.1 (95% CI, 25.0-27.2) fewer calories purchased in the 3- to 24-month follow-up period (Figure 2). Among non-California restaurants, however, we observed no association in the 3- to 24-month follow-up period (difference-in-differences, −1.8; 95% CI, −7.1 to 3.5).

**Table 3.** Difference-in-Differences Estimates of Calories Purchased per Transaction After Implementation of Menu Labeling, Overall and by Location

Variable	3- to 24-mo Follow-up period, average monthly effect size, β (95% CI)^a^		
	Difference, menu labeling restaurants^b^	Difference, comparison restaurants^c^	Difference-in-differences^d^
Overall	−0.8 (−2.9 to 1.3)	23.9 (22.8 to 24.9)	−24.7 (−25.7 to −23.6)
California^e^	9.8 (7.9 to 11.7)	35.9 (35.1 to 36.7)	−26.1 (−27.2 to −25.0)
Non-California^f^	−29.7 (−39.0 to −20.4)	−27.9 (−31.9 to −23.9)	−1.8 (−7.1 to 3.5)

^a^ Defined as the 3 to 24 months after the date of location-specific and setting-specific menu labeling.

^b^ Estimates represent the difference between the average calories purchased per transaction during the baseline period and the average calories purchased per transaction in the 3- to 24-month follow-up period for the menu labeling group (difference 1).

^c^ Estimates represent the difference between the average calories purchased per transaction during the baseline period and the average calories purchased per transaction in the 3- to 24-month follow-up period for the comparison group (difference 2).

^d^ Represents the difference between the differences for the menu labeling group and the comparison group, averaged over the 3- to 24-month follow-up period.

^e^ Excludes beverage data.

^f^ Includes beverage data from drive-through.

We observed a decrease in the total count of items purchased in the menu labeling vs comparison group in the 3- to 24-month follow-up period (eTable 4 in Supplement 1). The outcomes of menu labeling differed by food item category and time of day (eFigure 5 and eFigure 6 in Supplement 1). For example, the decrease in the total count of items purchased per restaurant-month was larger for tacos in the 3- to 24-month period (difference-in-differences, −723.4; 95% CI, −843.9 to −603.0) compared with other food items. Menu labeling of calories purchased during breakfast hours in the 3- to 24-month period (difference-in-differences, −67.4; 95% CI, 70.7 to −64.2) was larger than calories purchased in transactions made during other times of day, and we observed no association between calories purchased during late night hours in the 3- to 24-month period (difference-in-differences, −10.3; 95% CI, −43.4 to 22.8). We also observed that, among non-California restaurants, menu labeling was associated with fewer calories purchased in the drive-through setting (difference-in-differences, −13.7; 95% CI, −17.1 to −10.2) and yet more calories purchased in the in-store setting (difference-in-differences, 25.2; 95% CI, 17.9-32.5) vs the comparison group (eTable 4, eFigure 7 in Supplement 1).

In sensitivity analyses, we found that our results were robust to different baseline periods, the exclusion of new menu items, and the length of time restaurants were open following menu labeling implementation (eTable 5, eTable 6, and eFigure 8 in Supplement 1), indicating that restaurant closures were not associated with our findings. There was no statistically significant difference in the calorie count of the 100 highest-selling items sold in the baseline vs follow-up periods in the menu labeling and comparison group restaurants (eTable 7 in Supplement 1), suggesting our results were likely not associated with product reformulation (eg, reducing calorie count of popular items in response to menu labeling implementation).

## Discussion

Calorie labeling policies may have the potential to facilitate small behavioral changes in diets. In our study, we found that implementation of menu labeling in restaurants led to approximately 25 fewer calories purchased per transaction over a 2-year follow-up period compared with restaurants in areas with no such legislation. This finding differed by location and order setting, with fewer calories purchased in California, where menu labeling was only implemented in the interior of restaurants, and also in drive-through transactions in non-California restaurants and, in contrast, relatively more calories purchased inside of non-California restaurants in the menu labeling group. This could reflect differences in customer responses by region, which differ in terms of customer attitudes to calorie labels and order setting, where the presentation and awareness of calorie labels may differ. We plan to examine such differences in a future analysis by evaluating the national rollout of menu labeling legislation, when the implementation of menu labeling was extended to the drive-through setting of California restaurants. The lack of beverage data for in-store transactions may have also informed our results, since changes in beverage transactions in response to menu labeling may also differ by region.

In terms of food item categories, the changes observed with menu labeling appear to have been associated with fewer calories purchased from taco orders, the categories with the largest sales. Taco restaurants allow customers to customize orders, which could be one way customers reduced calories, in addition to reducing the type or number of items ordered. We also observed differences by time of day, with greater differences during breakfast hours, and no substantial change during late night hours. These results suggest that customers who purchase meals at off-peak times of day may differ from those who frequent the same restaurants during peak hours, potentially with different attitudes or perceptions about the energy density of meal options.

Two previous quasi-experimental studies analyzed sales data with mixed results.^8,25^ Using retail data from 14 Taco Time locations in King County, Washington, Finkelstein et al^25^ found that calories purchased did not change after calorie information was posted on drive-through menu boards. In contrast, Bollinger et al^8^ used Starbucks data to analyze menu labeling in New York City and found customers ordered 15 fewer calories in the year after implementation, which is consistent with our results. Both studies, however, were limited by their narrow geographic coverage and relatively short follow-up periods. Our results are also consistent with labeling in other settings, such as a workplace cafeteria labeling program (approximately 20 fewer calories purchased) and calorie labeling of prepared foods in supermarkets (eg, approximately 10-18 fewer calories purchased).^31,32^ In a pair of studies using an interrupted time-series design, Petimar et al^10,11^ analyzed sales data from 3 chain restaurants in the southern US and found that labeling was associated with a decrease in calories purchased 2 years after franchise labeling and 1 year after nationwide labeling (approximately 43-73 calories), which is relatively larger than our effect sizes. These studies, however, did not have comparison groups, which makes it difficult to eliminate potential bias.

### Limitations

Our study has several limitations. We lacked data related to food intake and were not able to observe the number of people per transaction. Other work, though, has shown that virtually all food purchased in fast food restaurants is consumed.^18^ These limitations are also a necessary trade-off of having a rich and large set of objective data. Another weakness was that we could not determine the type or number of refills for self-serve fountain beverages, and thus excluded beverage purchases from eat-in and take-out orders. Our matching approach may be biased if comparison restaurants differed in unobserved ways related to menu labeling.

## Conclusions

Our analysis adds to a growing body of evidence that suggests people adjust their food choices based on calorie labels. Small decreases in calories purchased could lead to small population-level improvements in health,^33^ especially in combination with other types of healthy eating policies (eg, nutrition warning labels), and labeling across multiple types of retailers may lead to even larger decreases in daily calories purchased over an extended period. In the future, it will be important to consider additional distributional outcomes of menu labeling legislation to estimate whether the policy impact is greater among subgroups of the population. Previous work, for example, suggests that outcomes associated with menu labeling may differ by age group and income.^10,34^ It will also be necessary to examine the differential impact of menu labeling by restaurant setting (eg, sit-down restaurants), where the use of calorie information may differ, and whether changes to the design and placement of calorie information provide additional benefits.

## Notes

Supplement 1. **eTable 1.** Number of Restaurants Open in Menu Labeling Group and Comparison Group, by Location and Months Open After Menu Labeling **eTable 2.** Restaurant-Level and Community-Level Characteristics of Menu Labeling and Comparison Restaurants, Overall and by Location, Unweighted and Weighted **eTable 3.** Difference-in-Differences Estimates of Calories Purchased per Transaction After Implementation of Menu Labeling, by Month **eTable 4.** Difference-in-Differences Model Estimates of Calories and Count of Items Purchased per Transaction After Implementation of Menu Labeling, by Location, Item Category, Time of Day, Nutrients, and Order Setting **eTable 5.** Difference-in-Differences Model Estimates of Calories Purchased per Transaction After Implementation of Menu Labeling, Sensitivity Analyses **eTable 6.** Difference-in-Differences Model Estimates of Calories Purchased per Transaction After Implementation of Menu Labeling, by Months Open After Menu Labeling **eTable 7.** Change in Calories of Top 100 High-Selling Items in Menu Labeling Group and Comparison Group **eFigure 1.** Map of the Locations of the Restaurants in the Menu Labeling Group and Comparison Group in the Final Sample **eFigure 2.** Percentage Sales by Food Category, Menu Labeling Group, and Comparison Group Combined **eFigure 3.** Percentage Sales by Time of Day, Menu Labeling Group, and Comparison Group Combined **eFigure 4.** Difference-in-Differences Model Estimates, by Absolute Nutrient Content **eFigure 5.** Difference-in-Differences Model Estimates, by Food Category **eFigure 6.** Difference-in-Differences Model Estimates, by Time of Day **eFigure 7.** Difference-in-Differences Model Estimates, by Order Setting **eFigure 8.** Difference-in-Differences Model Estimates of Calories Purchased per Transaction After Implementation of Menu Labeling, by Months Open After Menu Labeling **eMethods.** Supplementary Description of Matching Procedures and Statistical Analyses

Supplement 2.
Data Sharing Statement
