# Supplementary material for: Menu Labeling and Calories Purchased in Restaurants in a US National Fast Food Chain
Source: JAMA Netw Open. 2023 Dec 15;6(12):e2346851. doi: 10.1001/jamanetworkopen.2023.46851 (PMC10724762; doi:10.1001/jamanetworkopen.2023.46851)
Supplement: Supplement 1. — eTable 1. Number of Restaurants Open in Menu Labeling Group and Comparison Group, by Location and Months Open After Menu Labeling eTable 2. Restaurant-Level and Community-Level Characteristics of Menu Labeling and Comparison Restaurants, Overall and by Location, Unweighted and Weighted eTable 3. Difference-in-Differences Estimates of Calories Purchased per Transaction After Implementation of Menu Labeling, by Month eTable 4. Difference-in-Differences Model Estimates of Calories and Count of Items Purchased per Transaction After Implementation of Menu Labeling, by Location, Item Category, Time of Day, Nutrients, and Order Setting eTable 5. Difference-in-Differences Model Estimates of Calories Purchased per Transaction After Implementation of Menu Labeling, Sensitivity Analyses eTable 6. Difference-in-Differences Model Estimates of Calories Purchased per Transaction After Implementation of Menu Labeling, by Months Open After Menu Labeling eTable 7. Change in Calories of Top 100 High-Selling Items in Menu Labeling Group and Comparison Group eFigure 1. Map of the Locations of the Restaurants in the Menu Labeling Group and Comparison Group in the Final Sample eFigure 2. Percentage Sales by Food Category, Menu Labeling Group, and Comparison Group Combined eFigure 3. Percentage Sales by Time of Day, Menu Labeling Group, and Comparison Group Combined eFigure 4. Difference-in-Differences Model Estimates, by Absolute Nutrient Content eFigure 5. Difference-in-Differences Model Estimates, by Food Category eFigure 6. Difference-in-Differences Model Estimates, by Time of Day eFigure 7. Difference-in-Differences Model Estimates, by Order Setting eFigure 8. Difference-in-Differences Model Estimates of Calories Purchased per Transaction After Implementation of Menu Labeling, by Months Open After Menu Labeling eMethods. Supplementary Description of Matching Procedures and Statistical Analyses [file jamanetwopen-e2346851-s001.pdf]

Supplementary Online Content

Rummo PE, Mijanovich T, Wu E, et al. Menu labeling and calories purchased in restaurants in a US national fast food chain. *JAMA Netw Open*. 2023;6(12):e2346851. doi:10.1001/jamanetworkopen.2023.46851

eTable 1. Number of Restaurants Open in Menu Labeling Group and Comparison Group, by Location and Months Open After Menu Labeling

eTable 2. Restaurant-Level and Community-Level Characteristics of Menu Labeling and Comparison Restaurants, Overall and by Location, Unweighted and Weighted

eTable 3. Difference-in-Differences Estimates of Calories Purchased per Transaction After Implementation of Menu Labeling, by Month

eTable 4. Difference-in-Differences Model Estimates of Calories and Count of Items Purchased per Transaction After Implementation of Menu Labeling, by Location, Item Category, Time of Day, Nutrients, and Order Setting

eTable 5. Difference-in-Differences Model Estimates of Calories Purchased per Transaction After Implementation of Menu Labeling, Sensitivity Analyses

eTable 6. Difference-in-Differences Model Estimates of Calories Purchased per Transaction After Implementation of Menu Labeling, by Months Open After Menu Labeling

eTable 7. Change in Calories of Top 100 High-Selling Items in Menu Labeling Group and Comparison Group

eFigure 1. Map of the Locations of the Restaurants in the Menu Labeling Group and Comparison Group in the Final Sample

eFigure 2. Percentage Sales by Food Category, Menu Labeling Group, and Comparison Group Combined

eFigure 3. Percentage Sales by Time of Day, Menu Labeling Group, and Comparison Group Combined

eFigure 4. Difference-in-Differences Model Estimates, by Absolute Nutrient Content

eFigure 5. Difference-in-Differences Model Estimates, by Food Category

eFigure 6. Difference-in-Differences Model Estimates, by Time of Day

eFigure 7. Difference-in-Differences Model Estimates, by Order Setting

eFigure 8. Difference-in-Differences Model Estimates of Calories Purchased per Transaction After Implementation of Menu Labeling, by Months Open After Menu Labeling

eMethods. Supplementary Description of Matching Procedures and Statistical Analyses

This supplementary material has been provided by the authors to give readers additional information about their work.

© 2023 Rummo PE et al. *JAMA Network Open.*

eTABLE 1. Number of restaurants open in menu labeling group and comparison group, by location and months open after menu labeling

	Restaurants, open ever (n), unmatched	Restaurants with data available in baseline period (n), matched, open[a] ever	Restaurants with data available in baseline period (n (%)), matched, open ≥6 months	Restaurants with data available in baseline period (n (%)), matched, open ≥12 months	Restaurants with data available in baseline period (n (%)), matched, open ≥18 months	Restaurants with data available in baseline period (n (%)), matched, open ≥24 months
Menu labeling restaurants, all	474	474	463 (97.7%)	396 (83.5%)	371 (78.3%)	261 (55.1%)
Comparison restaurants, all[b]	11451	474	463 (97.7%)	401.6 (84.7%)	379.2 (80%)	268.9 (56.7%)
California	450	450	439 (97.6%)	378 (84%)	356 (79.1%)	246 (54.7%)
Comparison restaurants	1866	450	439 (97.6%)	378 (84%)	356 (79.1%)	246 (54.7%)
King County, WA	3	3	3 (100%)	1.5 (50%)	0 (0%)	0 (0%)
Comparison restaurants	2260	3	3 (100%)	2.8 (93.3%)	2.6 (86.7%)	2.45 (81.7%)
Suffolk County, NY	16	16	16 (100%)	14 (87.5%)	14 (87.5%)	14 (87.5%)
Comparison restaurants	1888	16	16 (100%)	16 (100%)	16 (100%)	16 (100%)
Schenectady County, NY	1	1	1 (100%)	1 (100%)	1 (100%)	1 (100%)
Comparison restaurants	1908	1	1 (100%)	1 (100%)	1 (100%)	1 (100%)
Montgomery County, MD	3	3	3 (100%)	3 (100%)	3 (100%)	3 (100%)
Comparison restaurants	1866	3	3 (100%)	3 (100%)	3 (100%)	3 (100%)
Vermont	1	1	1 (100%)	0 (0%)	0 (0%)	0 (0%)
Comparison restaurants	1666	1	1 (100%)	1 (100%)	1 (100%)	1 (100%)

[a]Open defined as a restaurant with transactions in each month in that period of months in the follow-up period.
[b]We used synthetic control methods to construct a comparison unit for each restaurant in the menu labeling group.

© 2023 Rummo PE et al. *JAMA Network Open.*

eTABLE 2. Restaurant-level and community-level characteristics of menu labeling and comparison restaurants, overall and by location, unweighted and weighted

	Unweighted		*Weighted*		
	Menu labeling restaurants	Comparison restaurants	Menu labeling restaurants	Comparison restaurants	Predictor weight
	Mean (SD)	Mean (SD)	Mean (SD)	Mean (SD)	
Restaurant-level characteristics					
Mean calories, total transactions, month -14	997 (131)	991 (128)	997 (131)	1043 (69)	0.042
Mean calories, total transactions, month -13	1055 (124)	1055 (123)	1055 (124)	1114 (53)	0.044
Mean calories, total transactions, month -12	1094 (123)	1097 (120)	1094 (123)	1159 (46)	0.044
Mean calories, total transactions, month -11	1132 (136)	1134 (130)	1132 (136)	1200 (51)	0.05
Mean calories, total transactions, month -10	1063 (136)	1069 (135)	1063 (136)	1131 (69)	0.047
Mean calories, total transactions, month -9	1064 (139)	1060 (139)	1064 (139)	1117 (78)	0.036
Mean calories, total transactions, month -8	1051 (132)	1052 (130)	1051 (132)	1107 (68)	0.034
Mean calories, total transactions, month -7	1041 (133)	1041 (130)	1041 (133)	1093 (72)	0.036
Mean calories, total transactions, month -6	1071 (131)	1070 (130)	1071 (131)	1125 (63)	0.045
Mean calories, total transactions, month -5	1020 (130)	1020 (130)	1020 (130)	1074 (71)	0.038
Mean calories, total transactions, month -4	1015 (139)	1022 (134)	1015 (139)	1077 (77)	0.048
Mean calories, total transactions, month -3	1012 (135)	1012 (130)	1012 (135)	1066 (72)	0.039
Drive-through transaction count, baseline period	7137 (3911)	6686 (3367)	7137 (3911)	6626 (2953)	0.024
Mean spending, drive-through transactions, baseline period	4.32 (0.98)	4.52 (0.6)	4.32 (0.98)	4.55 (0.44)	0.026
In-store transactions % (baseline period)	42.4 (18.6)	36.5 (10.2)	42.4 (18.6)	34.1 (8.5)	0.023
Lunch and dinner transactions % (baseline period)	59 (4.3)	59.5 (2.9)	59 (4.3)	58.7 (0.8)	0.025
Data available ≥18 months prior to menu labeling implementation	54.9 (49.8)	56.9 (31.6)	54.9 (49.8)	69 (10.7)	0.02
Data available ≥24 months prior to menu labeling implementation	50.2 (50.1)	56.2 (31.7)	50.2 (50.1)	68.5 (10.7)	0.019

© 2023 Rummo PE et al. *JAMA Network Open.*

Mean calories, drive-through transactions, trend	-9 (8)	-9 (6)	-9 (8)	-9 (2)	0.043
Drive-through transaction count, trend	-68 (335)	-35 (194)	-68 (335)	-26 (174)	0.025
Mean spending, drive-through transactions, baseline period	0.06 (0.03)	0.05 (0.02)	0.06 (0.03)	0.05 (0.01)	0.023
Community-level characteristics					
Greater than 65 years of age, %	19 (3.8)	18.9 (2.5)	19 (3.8)	19.3 (0.7)	0.024
Asian population, %	12 (12.7)	6.2 (5.9)	12 (12.7)	4.4 (2.6)	0.02
Black population, %	6.5 (9.3)	10.5 (8.2)	6.5 (9.3)	10.4 (2.1)	0.027
Income per capita	27660 (12475)	26412 (7081)	27660 (12475)	25514 (2422)	0.019
College degree or higher, %	17.5 (11.8)	19 (8.2)	17.5 (11.8)	17 (2.5)	0.025
Hispanic population, %	36.4 (23.1)	22.4 (13.3)	36.4 (23.1)	18.4 (5.6)	0.02
No high school degree, %	11.4 (7.4)	9.4 (4.4)	11.4 (7.4)	9.1 (1.6)	0.019
Male, %	49.3 (3.6)	48.9 (2.1)	49.3 (3.6)	48.6 (0.5)	0.027
Median household income	63006 (26149)	55722 (15341)	63006 (26149)	52601 (5262)	0.022
Population count	5491 (2306)	5339 (1339)	5491 (2306)	5115 (400)	0.027
Less than 18 years of age, %	17.9 (3.3)	17.6 (2.1)	17.9 (3.3)	17.4 (0.5)	0.026
White population, %	63.6 (19.1)	73.2 (11.6)	63.6 (19.1)	76.5 (4.6)	0.017

[a]We used synthetic control methods to construct a comparison unit for each restaurant in the menu labeling group.

© 2023 Rummo PE et al. *JAMA Network Open.*

eTABLE 3. Difference-in-differences estimates of calories purchased per transaction after implementation of menu labeling, by month

Month relative to menu labeling implementation time	Difference-in-differences[a], β (95% CI)
3	-23.2 (-23.2, -23.2)
4	-8.9 (-8.9, -8.9)
5	-21.4 (-21.5, -21.4)
6	-16.1 (-16.2, -16.1)
7	-18.1 (-18.2, -18.0)
8	-19.1 (-19.3, -18.9)
9	-29.8 (-30.0, -29.6)
10	-19.1 (-19.4, -18.9)
11	-18.5 (-18.7, -18.2)
12	-14.5 (-14.7, -14.3)
13	-20.3 (-20.6, -20.1)
14	-36.4 (-36.7, -36.2)
15	-26.9 (-27.2, -26.6)
16	-22.4 (-22.7, -22.1)
17	-31.3 (-31.6, -31.1)
18	-28.1 (-28.3, -27.8)
19	-23.4 (-23.6, -23.2)
20	-26.8 (-27.0, -26.6)
21	-37.8 (-38.0, -37.6)
22	-45.6 (-45.9, -45.3)
23	-22.3 (-22.7, -22.0)
24	-25.0 (-25.5, -24.5)

[a]Represents the difference between the differences in the average calories purchased per transaction for the menu labeling group and the comparison group for each month of the follow-up period.

© 2023 Rummo PE et al. *JAMA Network Open.*

eTABLE 4. Difference-in-differences model estimates of calories and count of items purchased per transaction after implementation of menu labeling, by location, item category, time of day, nutrients, and order setting

	Months 3-24, average monthly effect[a]		
	Difference[b], menu labeling restaurants	Difference[c], comparison restaurants	Difference-in-differences[d]
Overall	-0.8 (-2.9, 1.3)	23.9 (22.8, 24.9)	-24.6 (-25.7, -23.6)
Location			
California	9.8 (7.9, 11.7)	35.9 (35.1, 36.7)	-26.1 (-27.2, -25.0)
Non-California	-29.7 (-39.0, -20.4)	-27.9 (-31.9, -23.9)	-1.792 (-7.1, 3.5)
Item category[e]			
All items	-570.9 (-984.1, -157.7)	-396.4 (-596.5, -196.4)	-174.5 (-387.6, 38.7)
Taco	142.7 (-95.2, 380.7)	866.2 (748.7, 983.6)	-723.4 (-843.9, -603.0)
Burrito	-1744.6 (-1855.1, -1634.0)	-1344.5 (-1399.1, -1289.9)	-400 (-456, -344.1)
Salad	-527.7 (-565.8, -489.7)	-300.3 (-319.1, -281.6)	-227.4 (-246.7, -208.1)
Other entrée	-261.1 (-333.5, -188.7)	-619 (-654.8, -583.3)	357.9 (321.3, 394.6)
Desserts	-514.1 (-538.7, -489.4)	-651.9 (-664, -639.7)	137.8 (125.3, 150.2)
Beverages[f]	-980.1 (-1430.4, -529.8)	-711.4 (-907.2, -515.6)	-268.7 (-523.2, -14.2)
Time of day[g]			
Late night	7.5 (-47.4, 62.4)	17.9 (-3.9, 39.7)	-10.3 (-43.4, 22.8)
Breakfast	-51.5 (-58.2, -44.8)	15.9 (12.6, 19.3)	-67.4 (-70.7, -64.2)
Lunch	5.2 (3, 7.4)	17.8 (16.7, 18.9)	-12.6 (-13.7, -11.5)
Afternoon	2 (-0.7, 4.7)	23.3 (21.9, 24.7)	-21.3 (-22.6, -20.0)
Dinner	11.7 (8.7, 14.7)	35.7 (34.1, 37.2)	-24.0 (-25.5, -22.5)
Evening	8.5 (-13.8, 30.8)	39.5 (28.2, 50.8)	-31.0 (-42.0, -20.0)
Nutrients			
Total fat (g)	0.3 (0.2, 0.4)	1.3 (1.3, 1.4)	-1 (-1, -0.9)

© 2023 Rummo PE et al. *JAMA Network Open.*

Carbohydrates (g)	-1 (-1.2, -0.8)	2.2 (2.1, 2.3)	-3.2 (-3.3, -3.1)
Protein (g)	0.3 (0.2, 0.4)	1.4 (1.3, 1.4)	-1.1 (-1.1, -1.0)
Saturated fat (g)	0.5 (0.4, 0.5)	0.9 (0.9, 0.9)	-0.4 (-0.5, -0.4)
Sugar (g)	-0.5 (-0.6, -0.5)	-0.4 (-0.4, -0.4)	-0.2 (-0.2, -0.1)
Fiber (g)	0.3 (0.3, 0.4)	0.7 (0.7, 0.8)	-0.4 (-0.4, -0.4)
Sodium (mg)	-1.5 (-7.5, 4.6)	71.7 (68.7, 74.8)	-73.2 (-76.2, -70.2)
Order setting[h]			
In-store	-102.9 (-115.9, -90.0)	-128.1 (-133.7, -122.5)	25.2 (17.9, 32.5)
Drive-through	-0.3 (-6.3, 5.8)	13.4 (10.8, 16.1)	-13.7 (-17.1, -10.2)

[a]Defined as the 3 to 24 months after the date of location-specific and setting-specific menu labeling.
[b]Estimates represent the difference between the average calories purchased per transaction during the baseline period and the average calories purchased per transaction in the relevant year of the follow-up period for the menu labeling group (difference #1).
[c]Estimates represent the difference between the average calories purchased per transaction during the baseline period and the average calories purchased per transaction in the relevant year of the follow-up period for the comparison group (difference #2).
[d]Represents the difference between the differences for the menu labeling group and the comparison group, averaged over the 3-24 month follow-up period.
[e]Outcomes reflect count of items transacted per restaurant-month.
[f]Analyses with beverage data reflect drive-through transactions only.
[g]Late night (00:00-03:59), breakfast (04:00-10:59), lunch (11:00-13:59), afternoon (14:00-16:59), dinner (17:00-20:59), evening (21:00-23:59).
[h]Among non-California restaurants only.

© 2023 Rummo PE et al. *JAMA Network Open.*

eTABLE 5. Difference-in-differences model estimates of calories purchased per transaction after implementation of menu labeling, sensitivity analyses

	Months 3-24, average monthly effect[a]		
	Difference[b], menu labeling restaurants	Difference[c], comparison restaurants	Difference[d], menu labeling restaurants
	β (95% CI)	β (95% CI)	β (95% CI)
Unmatched[e]	2.0 (1.5, 2.6)	15.6 (15.6, 15.7)	-13.6 (-14.1, -13.1)
Matched[f]	-0.8 (-2.9, 1.3)	23.9 (22.8, 24.9)	-24.7 (-25.7, -23.6)
Matched, 10-month baseline[g]	-7.9 (-9.4, -6.3)	16.9 (16.2, 17.6)	-24.7 (-25.6, -23.9)
Matched, 12-month baseline[h]	-4.4 (-5.4, -3.4)	21.2 (20.9, 21.6)	-25.6 (-26.3, -25.0)
Matched, excluding new menu items[i]	-49.9 (-52.2, -47.6)	-23.6 (-24.7, -22.5)	-26.3 (-27.5, -25.1)

[a]Defined as the effect averaged over the specificed number of months after the date of location-specific and setting-specific menu labeling.
[b]Estimates represent the difference between the average calories purchased per transaction during the baseline period and the average calories purchased per transaction in the relevant year of the follow-up period for the menu labeling group (difference #1).
[c]Estimates represent the difference between the average calories purchased per transaction during the baseline period and the average calories purchased per transaction in the relevant year of the follow-up period for the comparison group (difference #2).
[d]Represents the difference between the differences for the menu labeling group and the comparison group, averaged over the relevant year of the follow-up period.
[e]Unmatched refers to an analysis with all potential restaurants in the comparison group, prior to synthetic control matching and weighting.
[f]Matched refers to the primary analytic approach of synthetic control matching and weighting (vs. unmatched), with the 3-8-month baseline period.
[g]The baseline period re-defined as 3-10 months prior to menu labeling implementation.
[h]The baseline period re-defined as 3-12 months prior to menu labeling implementation.
[i]New menu items are items that were never observed in the pre-baseline period; new menu items account for 4.1% of sales in the follow-up period.

© 2023 Rummo PE et al. *JAMA Network Open.*

eTABLE 6. Difference-in-differences model estimates of calories purchased per transaction after implementation of menu labeling, by months open after menu labeling

Month relative to menu labeling implementation time	Difference-in-differences[a], β (95% CI), ≤12 months	Difference-in-differences[a], β (95% CI), ≤15 months	Difference-in-differences[a], β (95% CI), ≤18 months	Difference-in-differences[a], β (95% CI), ≤21 months	Difference-in-differences[a], β (95% CI), ≤24 months
3	-23.455 (-23.459, -23.451)	-23.542 (-23.546, -23.538)	-23.715 (-23.72, -23.711)	-22.955 (-22.959, -22.95)	-19.548 (-19.548, -19.548)
4	-9.978 (-9.982, -9.974)	-10.285 (-10.289, -10.28)	-10.262 (-10.266, -10.257)	-9.574 (-9.579, -9.57)	-7.476 (-7.476, -7.476)
5	-22.386 (-22.389, -22.382)	-22.269 (-22.273, -22.266)	-22.506 (-22.509, -22.502)	-21.997 (-21.997, -21.997)	-21.556 (-21.556, -21.556)
6	-14.949 (-14.952, -14.946)	-15.083 (-15.087, -15.08)	-15.287 (-15.291, -15.284)	-14.869 (-14.869, -14.869)	-15.116 (-15.116, -15.116)
7	-19.334 (-19.341, -19.328)	-19.431 (-19.438, -19.425)	-19.509 (-19.516, -19.502)	-19.544 (-19.548, -19.54)	-21.11 (-21.11, -21.11)
8	-19.245 (-19.257, -19.232)	-19.471 (-19.477, -19.465)	-19.71 (-19.716, -19.704)	-19.696 (-19.7, -19.692)	-21.97 (-21.97, -21.97)
9	-29.679 (-29.705, -29.652)	-29.967 (-29.988, -29.947)	-30.093 (-30.102, -30.084)	-29.755 (-29.758, -29.751)	-31.548 (-31.548, -31.548)
10	-19.16 (-19.197, -19.123)	-19.181 (-19.209, -19.153)	-19.183 (-19.2, -19.167)	-18.789 (-18.797, -18.78)	-20.446 (-20.446, -20.446)
11	-18.543 (-18.588, -18.499)	-18.587 (-18.623, -18.551)	-18.343 (-18.364, -18.322)	-17.69 (-17.698, -17.681)	-16.462 (-16.462, -16.462)
12	-14.598 (-14.651, -14.546)	-14.366 (-14.41, -14.323)	-14.374 (-14.399, -14.35)	-13.519 (-13.519, -13.519)	-14.115 (-14.115, -14.115)
13	-20.399 (-20.47, -20.327)	-20.45 (-20.494, -20.406)	-20.395 (-20.42, -20.369)	-19.401 (-19.405, -19.398)	-19.353 (-19.353, -19.353)
14	-36.499 (-36.581, -36.416)	-36.355 (-36.41, -36.3)	-36.291 (-36.327, -36.255)	-35.848 (-35.856, -35.84)	-32.035 (-32.035, -32.035)
15	-26.987 (-27.088, -26.885)	-26.981 (-27.051, -26.912)	-26.94 (-26.99, -26.889)	-26.485 (-26.498, -26.472)	-22.565 (-22.565, -22.565)
16	-22.474 (-22.572, -22.376)	-22.468 (-22.534, -22.402)	-22.87 (-22.923, -22.818)	-22.396 (-22.403, -22.389)	-18.7 (-18.7, -18.7)
17	-31.438 (-31.516, -31.359)	-31.432 (-31.479, -31.385)	-31.786 (-31.824, -31.748)	-31.771 (-31.774, -31.768)	-29.483 (-29.483, -29.483)
18	-28.077 (-28.14, -28.014)	-27.822 (-27.86, -27.784)	-28.198 (-28.226, -28.17)	-28.274 (-28.282, -28.265)	-26.066 (-26.066, -26.066)
19	-23.372 (-23.437, -23.307)	-23.088 (-23.128, -23.048)	-23.492 (-23.521, -23.463)	-23.836 (-23.845, -23.828)	-20.925 (-20.925, -20.925)
20	-26.754 (-26.796, -26.712)	-26.465 (-26.485, -26.445)	-26.474 (-26.487, -26.461)	-26.443 (-26.447, -26.44)	-22.536 (-22.536, -22.536)
21	-37.769 (-37.81, -37.727)	-37.734 (-37.754, -37.714)	-37.935 (-37.948, -37.923)	-38.131 (-38.135, -38.127)	-33.035 (-33.035, -33.035)
22	-45.535 (-45.646, -45.424)	-45.443 (-45.533, -45.354)	-45.586 (-45.665, -45.506)	-45.412 (-45.483, -45.341)	-40.624 (-40.624, -40.624)
23	-22.44 (-22.638, -22.243)	-22.512 (-22.689, -22.335)	-22.561 (-22.73, -22.393)	-22.547 (-22.703, -22.391)	-17.985 (-17.985, -17.985)
24	-24.909 (-25.269, -24.548)	-25.04 (-25.382, -24.697)	-25.372 (-25.708, -25.036)	-25.512 (-25.843, -25.181)	-23.886 (-23.886, -23.886)

[a]Represents the difference between the differences in the average calories purchased per transaction for the menu labeling group and the comparison group for each month of the follow-up period.

© 2023 Rummo PE et al. *JAMA Network Open.*

eTABLE 7. Change in calories of top 100 high-selling items[a] in menu labeling group and comparison group

	Baseline period[b]	Follow-up period[c]	
	Mean (SD)	Mean (SD)	p-value[d]
Among top 100 high-selling items that were sold in each period, menu labeling group[e]	329 (199)	323 (195)	0.83
Among top 100 high-selling items that were sold in each period, comparison group[e]	315 (191)	331 (209)	0.57

[a]The top 100 highest-selling items account for 97.1% of all transactions during the study period.
[b]Defined as the three to eight months prior to the date of location-specific and setting-specific menu labeling.
[c]Defined as the three to twelve months after the date of location-specific and setting-specific menu labeling.
[d]Estimated using an equality test.
[e]An item is eligible for this analysis if it was sold at least once every month between the start of the baseline period to the end of the follow-up period.

© 2023 Rummo PE et al. *JAMA Network Open.*

eFIGURE 1. Map of the locations of the restaurants in the menu labeling group and comparison group in the final sample

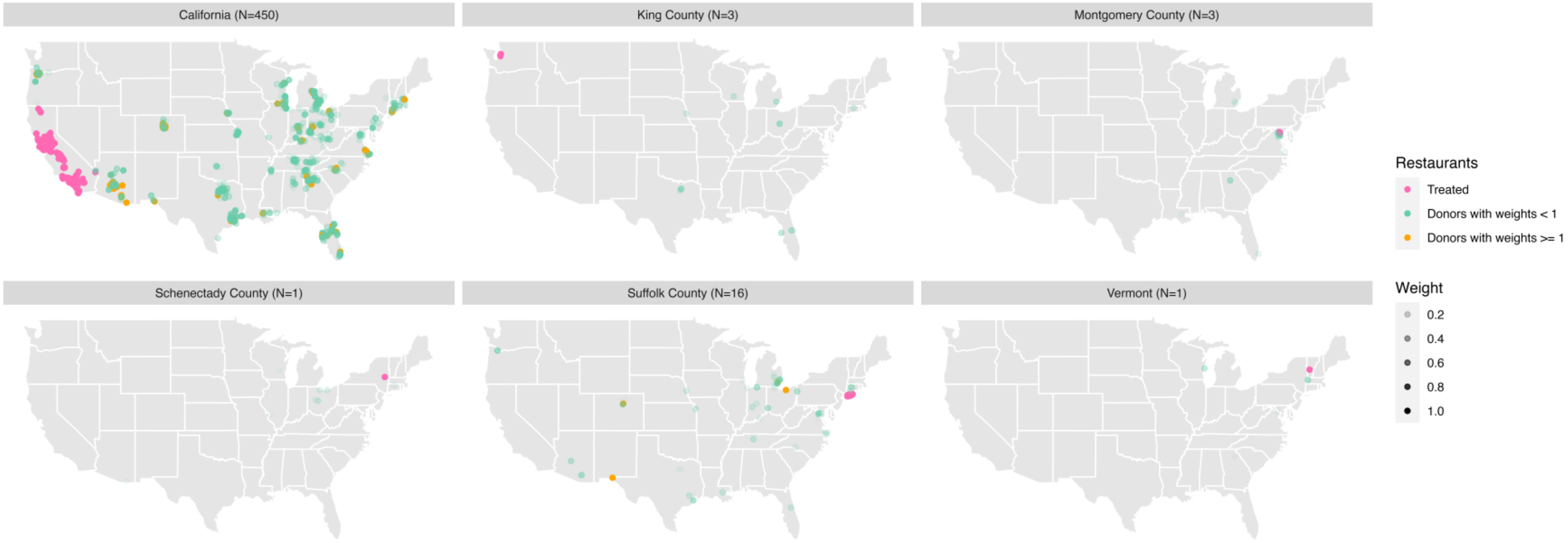

NOTES: There is no geographic overlap between treated and comparison units, and thus no potential for spillover, despite the seeming close proximity of treated and comparison units on some parts of the map (e.g., Montgomery County).

© 2023 Rummo PE et al. *JAMA Network Open.*

eFIGURE 2. Percentage sales by food category,[a] menu labeling group and comparison group combined

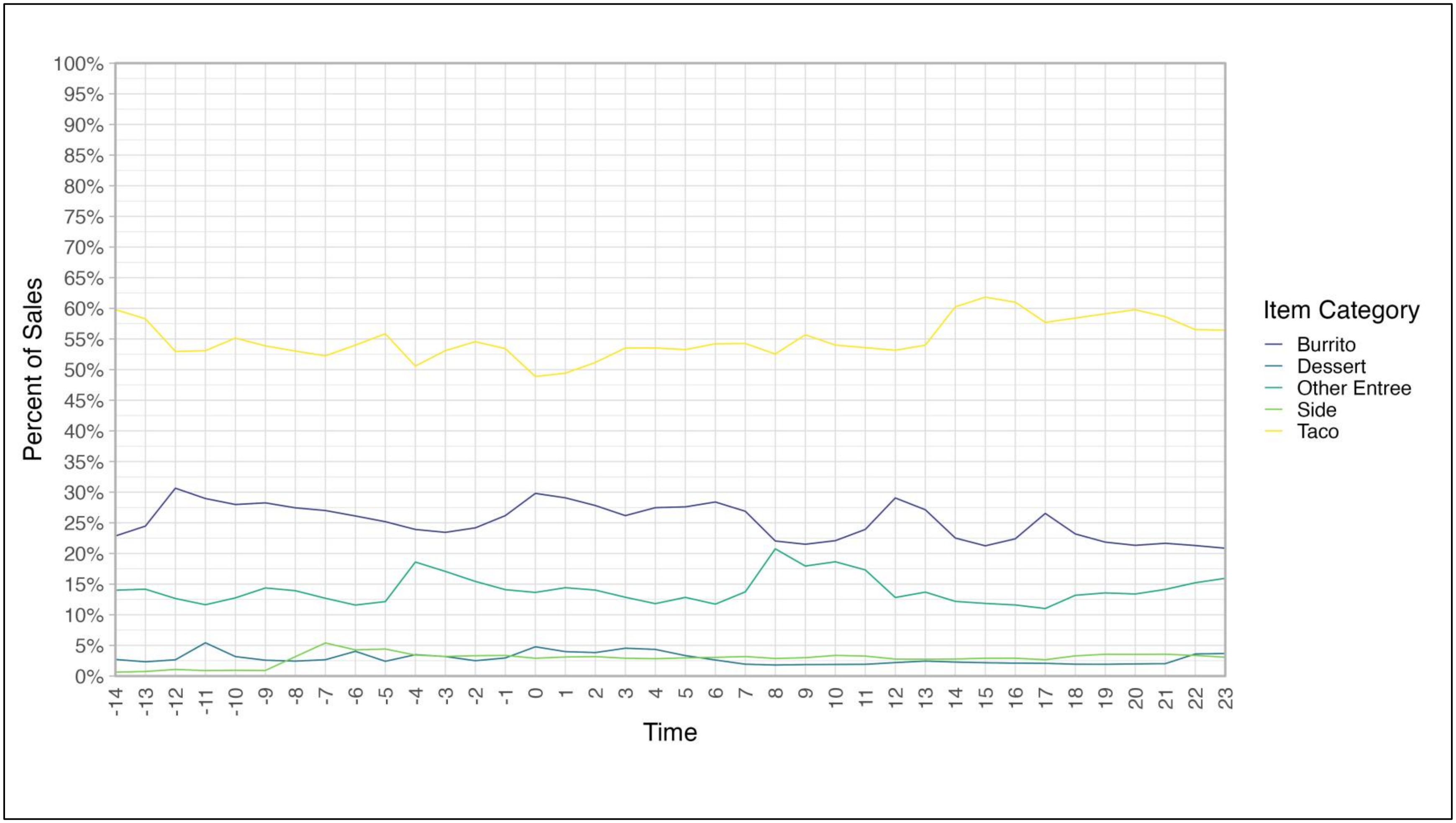

[a]Excluding beverage data.

© 2023 Rummo PE et al. *JAMA Network Open.*

eFIGURE 3. Percentage sales by time of day,[a] menu labeling group and comparison group combined

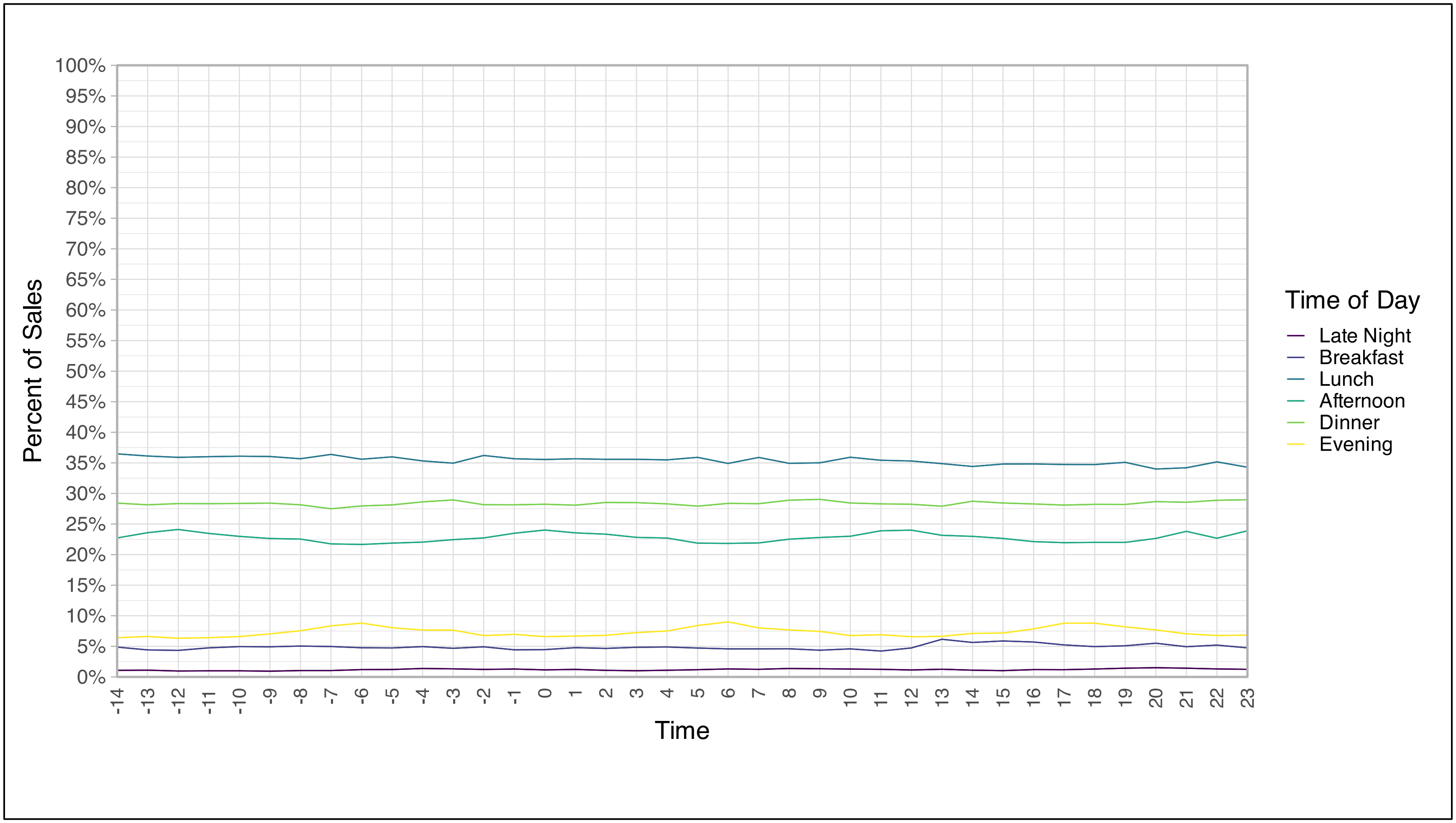

[a]Late night (00:00-03:59), breakfast (04:00-10:59), lunch (11:00-13:59), afternoon (14:00-16:59), dinner (17:00-20:59), evening (21:00-23:59).

© 2023 Rummo PE et al. *JAMA Network Open.*

eFIGURE 4. Difference-in-differences model estimates, by absolute nutrient content

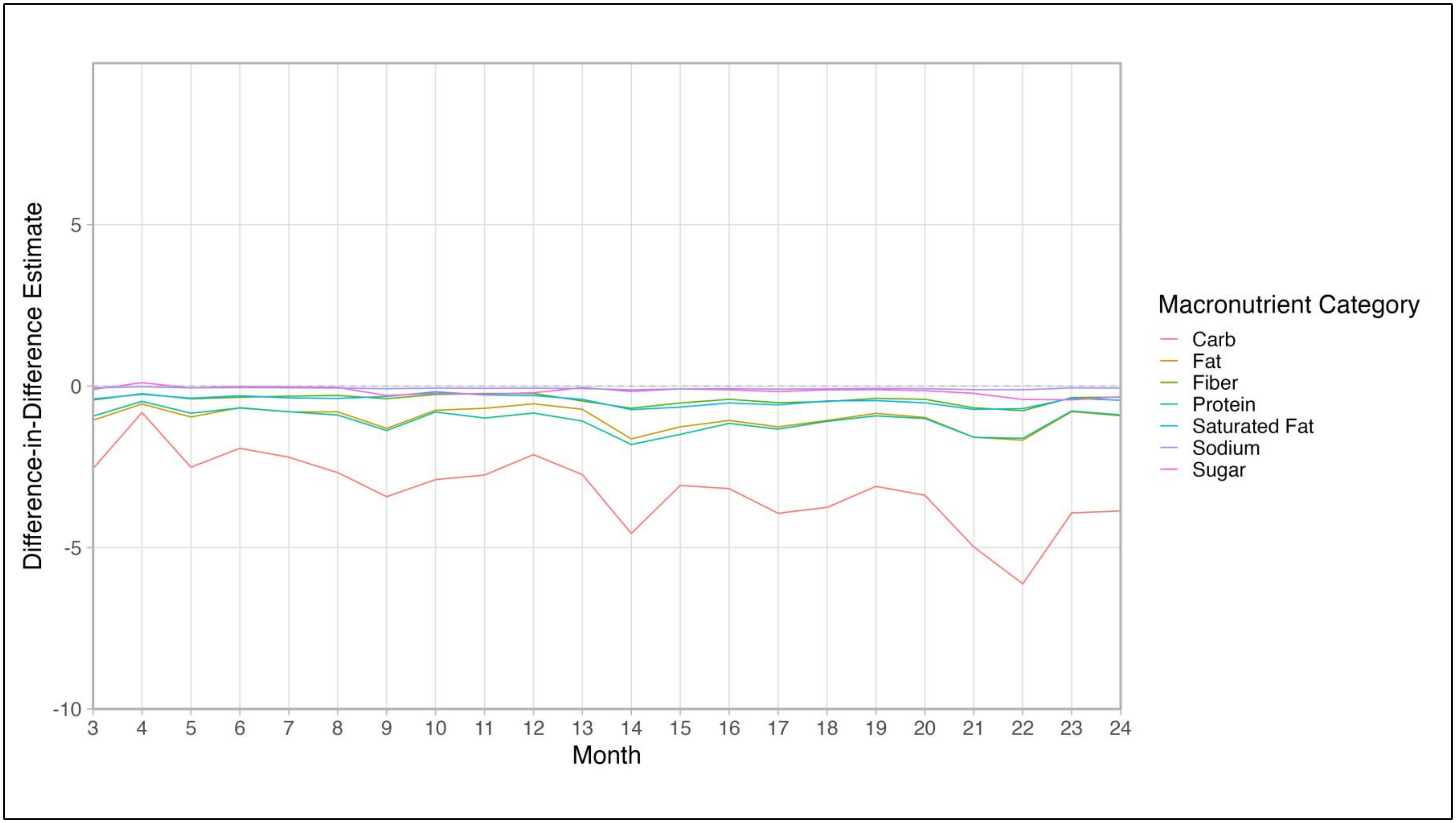

NOTES: Carb=Carbohydrates

© 2023 Rummo PE et al. *JAMA Network Open.*

eFIGURE 5. Difference-in-differences model estimates,[a] by food category

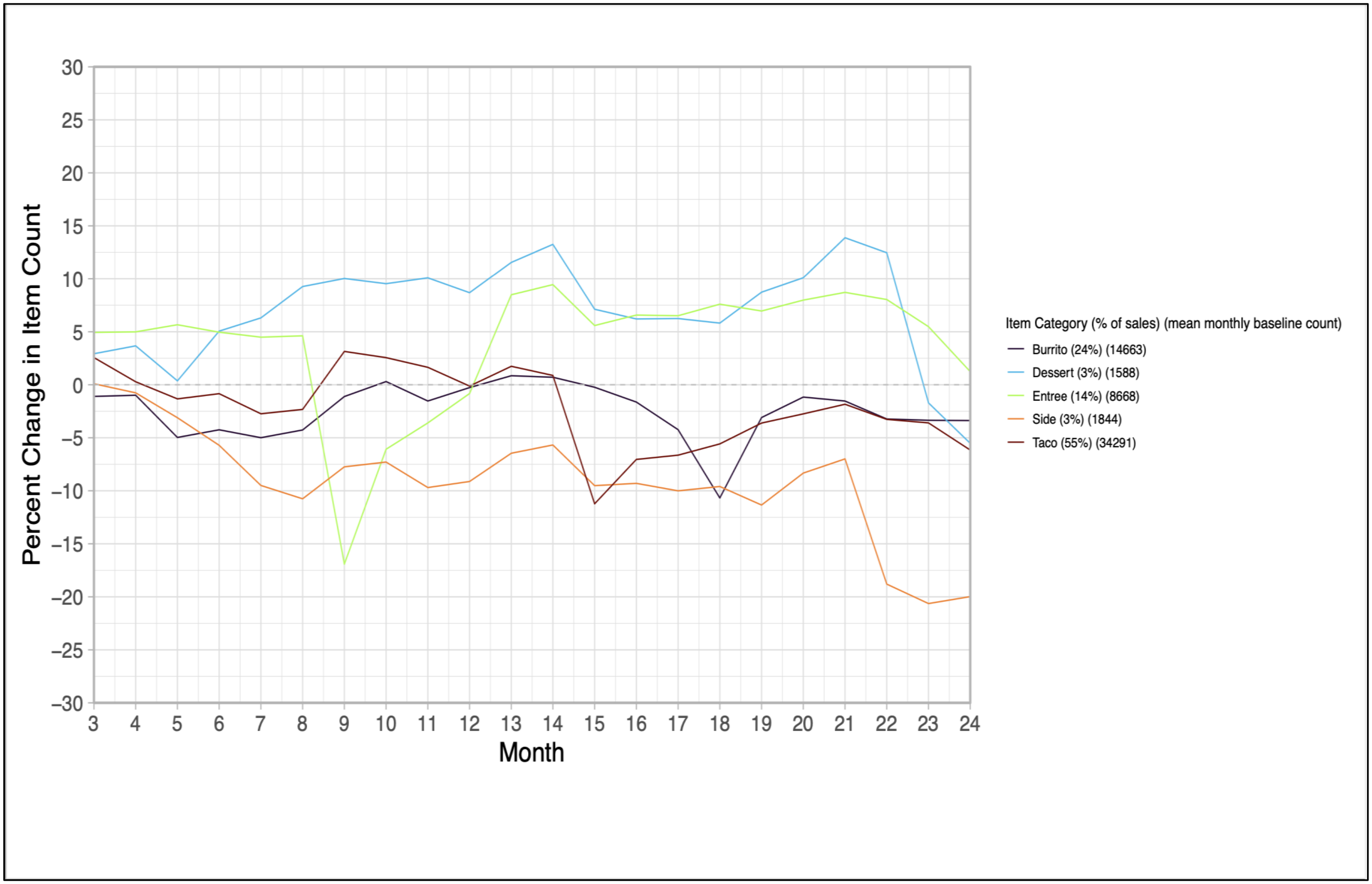

[a]Excluding beverage data.

© 2023 Rummo PE et al. *JAMA Network Open.*

eFIGURE 6. Difference-in-differences model estimates, by time of day[a]

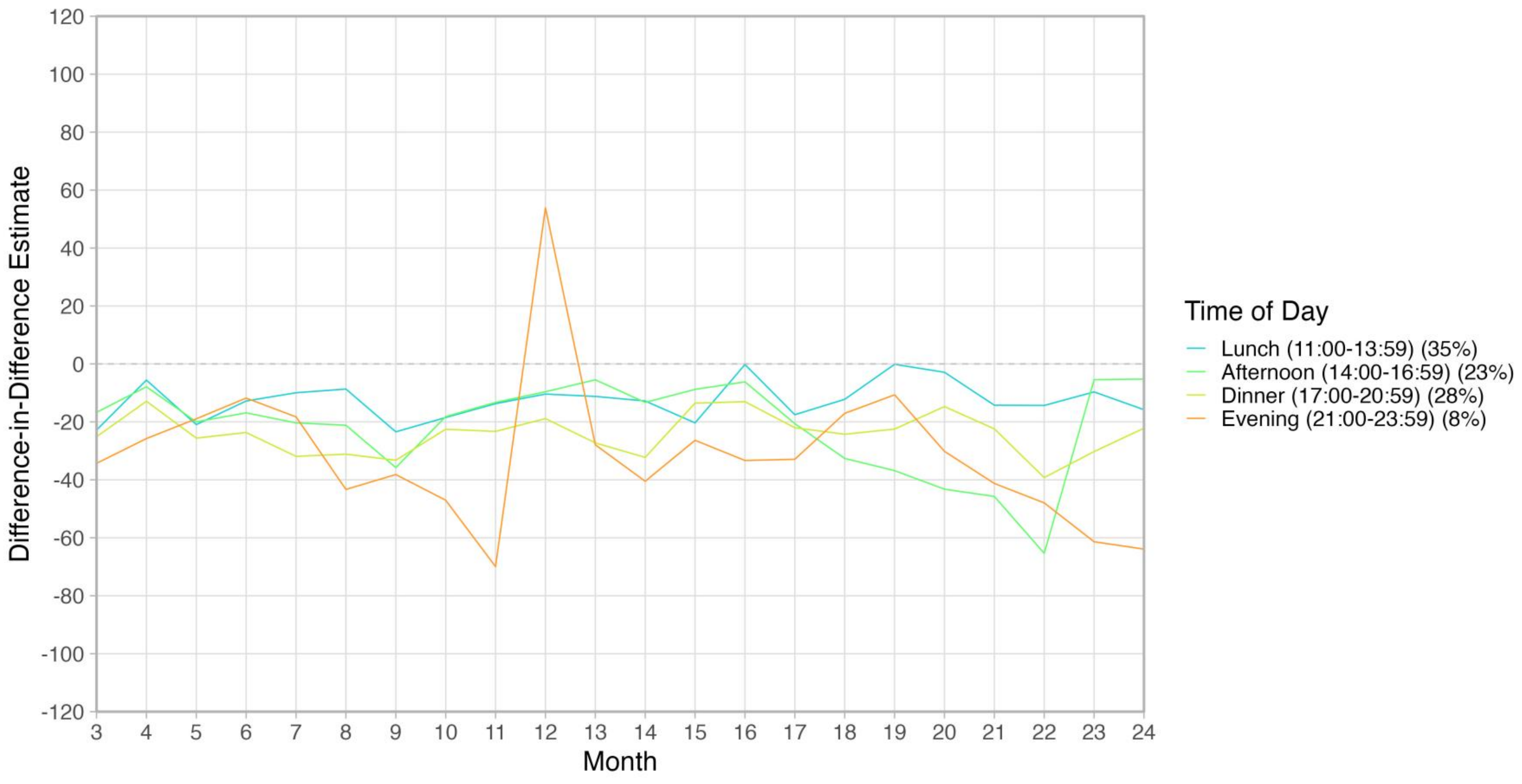

[a]Late night (00:00-03:59), breakfast (04:00-10:59), lunch (11:00-13:59), afternoon (14:00-16:59), dinner (17:00-20:59), evening (21:00-23:59).

© 2023 Rummo PE et al. *JAMA Network Open.*

eFIGURE 7. Difference-in-differences model estimates, by order setting[a]

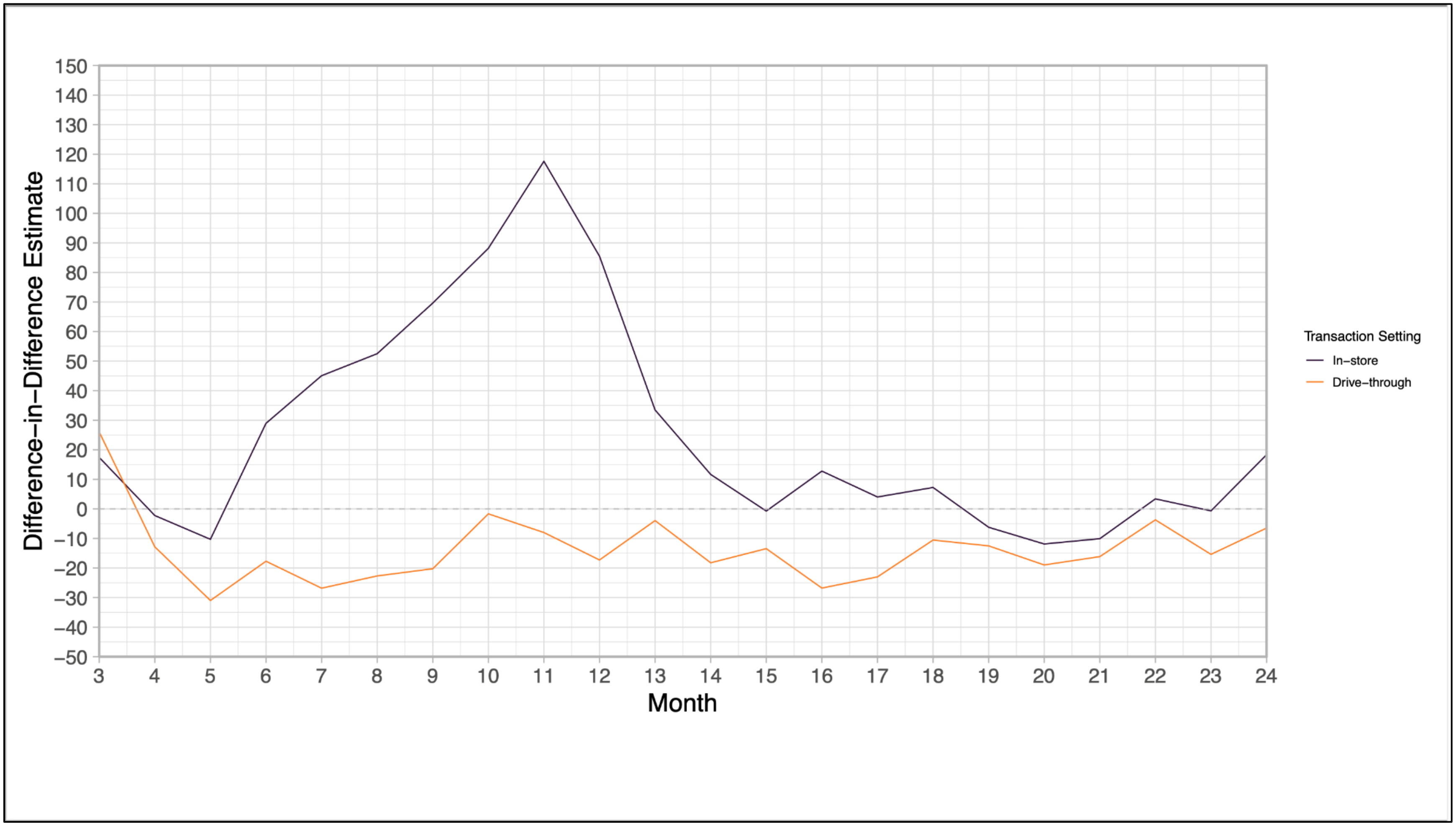

[a]Excluding beverage data in the in-store setting.

© 2023 Rummo PE et al. *JAMA Network Open.*

eFIGURE 8. Difference-in-differences model estimates of calories purchased per transaction after implementation of menu labeling, by months open after menu labeling

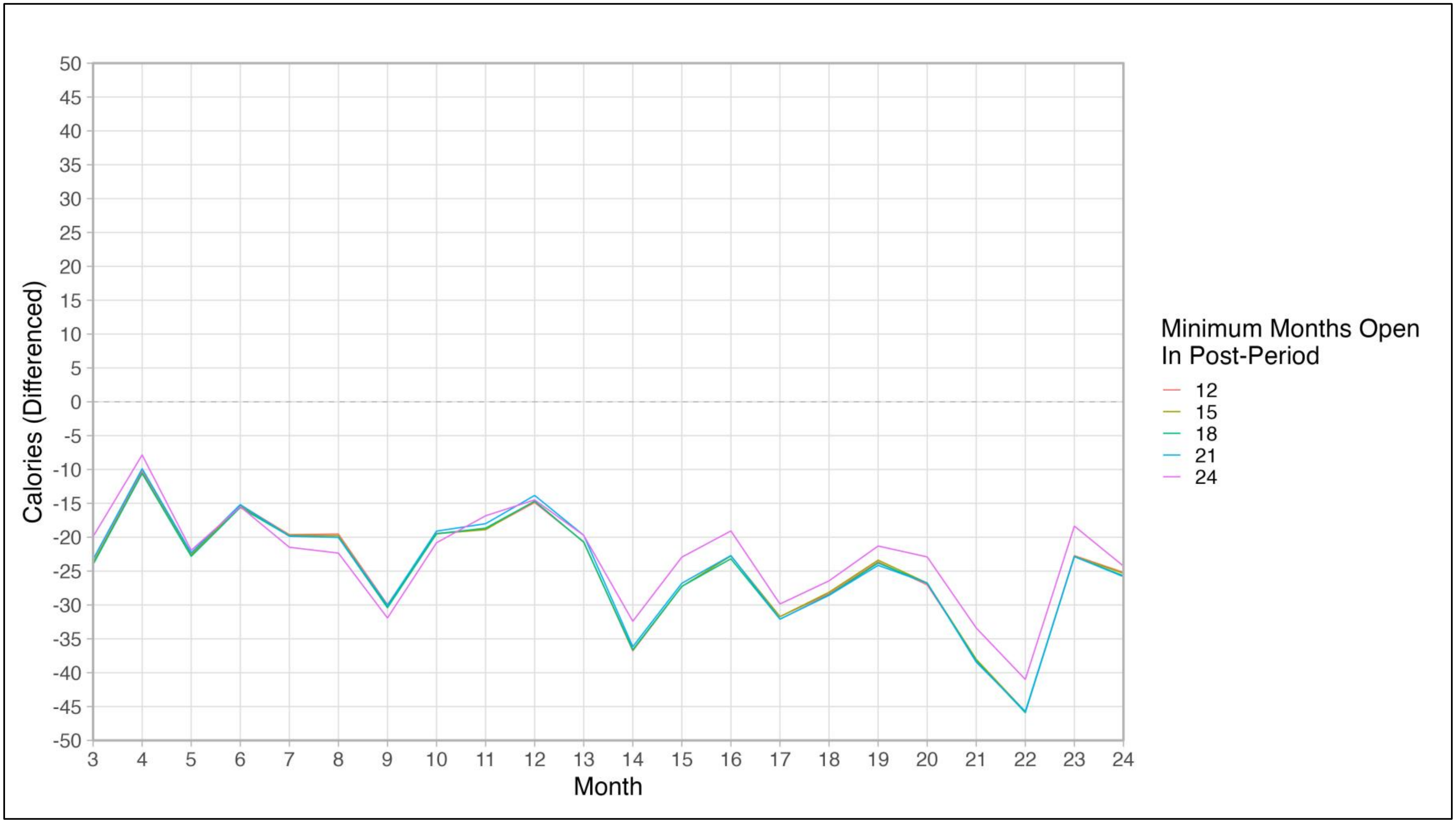

© 2023 Rummo PE et al. *JAMA Network Open.*

eMethods. Supplementary description of matching procedures and statistical analyses

Sample size of menu labeling and comparison restaurants

© 2023 Rummo PE et al. *JAMA Network Open.*

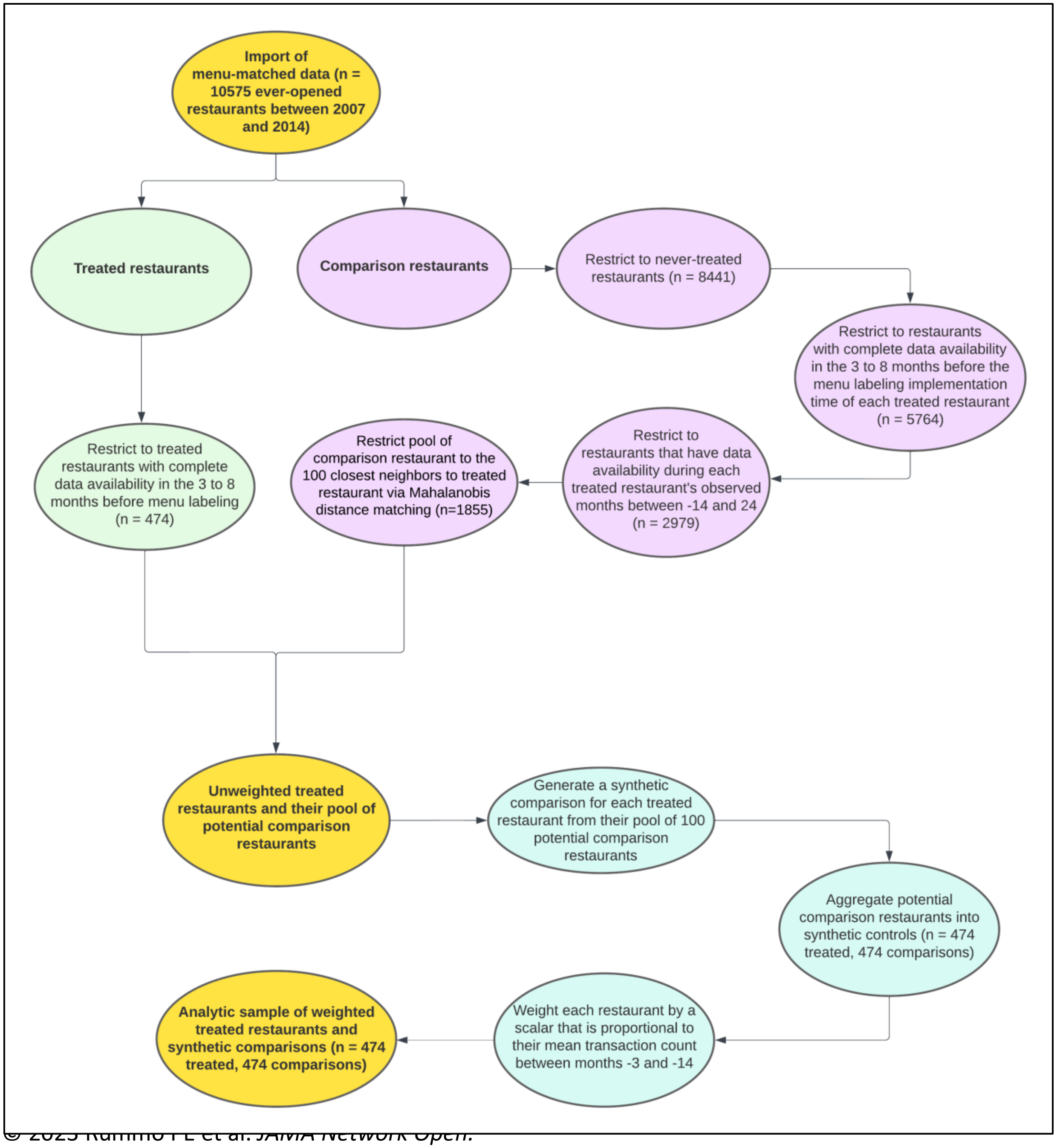

© 202[illegible] Rummo PE et al. *JAMA Network Open.*

NOTE: A comparison restaurant can serve as a comparison to multiple treated restaurants. Each synthetic control unit is comprised of 100 non-zero-weighted comparison restaurants, with weights summing to 1.

Code related to the matching procedures and the statistical analyses, including annotations
file:///C:/Users/rummop01/AppData/Local/Microsoft/Windows/INetCache/Content.Outlook/E4MTJOF7/codeshare.html

Statistical model

$$Y_{it} = \beta_0 + \beta_1 label_i + \beta_2 month_t + \sum_{j=-14, j\neq -3}^{24} \beta_{3j}(label_i \times month_t) + \alpha_k + \tau_i + \epsilon_i$$

Where:

- The estimand of interest is the vector of Average Treatment Effect on the Treated (ATT), β_{3j} or the monthly difference-in-difference coefficients relative to $t = -3$ (implementation month)
- i on $label_i$ denotes a treated or untreated restaurant
- t on $month_t$ denotes relative month from implementation, with 0 being the implementation month, presented as a vector of factor variables
- k on α_k denotes calendar months 1 through 12 for January to December (also presented as a vector of factor variables)
- Y denotes the outcome, i.e., mean calories per transaction in that specific restaurant-month
- The vector α_k denotes calendar month fixed effects to control for seasonality
- The vector τ_i denotes restaurant fixed effects to control for time-invariant confounding at the restaurant-level
- ϵ_i denotes the restaurant-level random error term

Methods for matching of menu items with nutrition information in MenuStat
Using the stringdist function in R, we calculated the Jaccard distance (i.e., degree of similarity in text strings) between items in our database and MenuStat and selected the most similar pair of items. To address ambiguity in non-exact matches (93%), five Research Assistants completed two rounds of manual item name matching. Each Research Assistant was assigned two lists of 1,000 items and instructed to confirm whether items were a match, not a match, or maybe a match. Each item was evaluated twice by two different Research Assistants independently, and inter-rater reliability was substantial (Cohen's weighted Kappa=0.71). Prior to arbitration, we filtered the dataset to the top 95% of sales for item pairs classified as not a match or maybe a match. For item pairs classified as maybe a match, a third Research Assistant classified the items as a match or not a match. For all item pairs classified as not a match by at least two Research Assistants, a manual search of similar items on MenuStat and other internet sources was undertaken.

© 2023 Rummo PE et al. *JAMA Network Open.*
