# Supplementary material for: Menu Labeling and Calories Purchased in Restaurants in a US National Fast Food Chain
Source: JAMA Netw Open. 2023 Dec 15;6(12):e2346851. doi: 10.1001/jamanetworkopen.2023.46851 (PMC10724762; doi:10.1001/jamanetworkopen.2023.46851)
Supplement: Supplement 2. — Data Sharing Statement [file jamanetwopen-e2346851-s002.pdf]

# Data Sharing Statement

Rummo. Menu Labeling and Calories Purchased in Restaurants in a US National Fast Food Chain. *JAMA Netw Open*. Published December 11, 2023.
doi:10.1001/jamanetworkopen.2023.46851

**Data**
**Data available:** No

**Additional Information**
**Explanation for why data not available:** The data are proprietary.
